# High dopamine impairs early neuronal identity and morphology in human hippocampal progenitor cells

**DOI:** 10.3389/fcell.2026.1783035

**Published:** 2026-03-30

**Authors:** Miryam Ravji, Gargi Mandal, Lucia Batzu, Sandrine Thuret

**Affiliations:** Basic & Clinical Neuroscience Department, Institute of Psychiatry, Psychology & Neuroscience, King’s College London, London, United Kingdom

**Keywords:** adult hippocampal neurogenesis, dopamine, dopamine receptors, human hippocampal progenitor cells, in vitro human model, neural stem cells, neuronal differentiation

## Abstract

Adult hippocampal neurogenesis (AHN) supports learning, memory, and emotional regulation, and is regulated by intrinsic and extrinsic factors. Dopamine influences neurogenesis in animal models, but its direct effects on human hippocampal progenitors and receptor-specific mechanisms remain unclear. This study examined the dose-dependent effects of dopamine on proliferation, differentiation, and survival of human hippocampal progenitor cells (HPC0A07/03) *in vitro*, and assessed dopamine D4 receptor (DRD4) involvement. Cells were treated with dopamine (1–150 µM) under proliferation and differentiation conditions, with DRD4 modulated via selective agonist, antagonist, or combined treatment. Proliferation (Ki67), stemness (SOX2, Nestin), neuronal differentiation (DCX, MAP2), apoptosis (CC3), total cell counts, and morphology (cytoplasmic area) were assessed using immunocytochemistry, alongside targeted gene expression analysis of cellular stress- and neurogenesis-related pathways. Treatment with supraphysiological dopamine concentration (150 µM) significantly reduced cell counts during differentiation and decreased SOX2 expression during proliferation, suggesting impaired survival and reduced stemness. Complementary transcriptional changes supported a stress-associated cellular response at high dopamine concentrations. Elevated dopamine (150 µM) also increased cytoplasmic area in immature DCX+ neurons during the differentiation phase, suggesting altered morphological maturation. Moderate dopamine concentration (30 µM) showed a trend toward increased proliferation and higher cell counts. No significant changes occurred for other markers or following DRD4 modulation. These findings indicate that dopamine’s effects on human hippocampal progenitors are dose-dependent: supraphysiological levels may compromise survival and progenitor identity, potentially via stress-related mechanisms, whereas moderate levels may support neurogenic processes. Understanding this dose-dependent balance has implications for neurological and psychiatric disorders involving dopaminergic dysregulation.

## 1 Introduction

Adult hippocampal neurogenesis (AHN) is the generation of new granule neurons from neural stem and progenitor cells (NSPCs) within the subgranular zone of the dentate gyrus of the hippocampus. Although once thought incapable of neurogenesis, the adult mammalian brain is now known to exhibit AHN in rodents, with converging post-mortem, transcriptomic, and computational evidence supporting its presence in humans (Eriksson et al., 1998; Spalding et al., 2013; Kuhn et al., 2016; Kempermann, 2022; Zhou et al., 2022; Dumitru et al., 2025). Nevertheless, the extent and functional relevance of AHN in the adult human hippocampus remain debated, partly due to conflicting post-mortem findings and technical limitations (Lee and Thuret, 2018; Sorrells et al., 2018; Moreno-Jiménez et al., 2019). These uncertainties highlight the need for human-relevant models to investigate mechanisms regulating neurogenesis.

Within the neurogenic niche, radial glia-like type 1 stem cells generate transient progenitors, neuroblasts, and ultimately mature granule neurons that integrate into hippocampal circuits over several weeks (Gonçalves et al., 2016; Kempermann, 2022). During early maturation, adult-born neurons exhibit heightened excitability and synaptic plasticity, distinguishing them from developmentally generated neurons (Brunner et al., 2014; Cole et al., 2020), and they contribute to hippocampal-dependent processes such as pattern separation, spatial memory encoding, and adaptive learning (Sahay et al., 2011; Frechou et al., 2024).

AHN is regulated by intrinsic and extrinsic factors that shape NSPC behaviour across proliferation, differentiation, and integration (Jiang et al., 2023). Intrinsic regulators include transcription factors and epigenetic mechanisms, such as DNA methylation and histone modification, that govern lineage commitment and neuronal maturation (Beckervordersandforth et al., 2015; Sheehy et al., 2022). Disruption of these mechanisms can impair distinct stages of neurogenesis; for example, deletion of *MECP2* compromises neuronal integration, while loss of *Mll1* interferes with neuronal fate specification (Lim et al., 2009; Sun et al., 2019). Extrinsic modulators, including physical activity, diet, stress, aging, and systemic inflammation, also shape the neurogenic niche (Stangl and Thuret, 2009; Jiang et al., 2023; Farmand et al., 2025). Exercise and environmental enrichment enhance proliferation and differentiation, partly through upregulation of neurotrophic factors including brain-derived neurotrophic factor (BDNF) (Garrett et al., 2012; Nokia et al., 2016). In contrast, chronic stress and aging suppress AHN by reducing progenitor proliferation and dendritic complexity of newborn neurons, contributing to diminished hippocampal plasticity (Shin et al., 2024; Finch et al., 2025).

Neurotransmitters further regulate AHN in an activity-dependent manner. While serotonergic and GABAergic regulation of AHN has been extensively characterised (Santarelli et al., 2003; Song et al., 2012), the role of dopamine remains comparatively underexplored. Dopaminergic neurons from the ventral tegmental area project to the hippocampus, where dopamine modulates synaptic plasticity and memory encoding (Lisman and Grace, 2005; Morales and Margolis, 2017; Sayegh et al., 2024). Dopamine signals via D1-like (D1/D5) and D2-like (D2–D4) receptors, which stimulate or inhibit cyclic AMP signalling, respectively (Beaulieu and Gainetdinov, 2011; Tritsch and Sabatini, 2012). These pathways are well characterised in mature neurons but remain poorly defined in NSPCs, particularly in the human hippocampus (Speranza et al., 2021).

Experimental evidence suggests that dopamine influences AHN in a dose- and receptor-dependent manner. Activation of D1-like receptors has been associated with increased progenitor proliferation and neuronal survival in rodent models and *in vitro* systems (Takamura et al., 2014; Mishra et al., 2019). In contrast, D2-like receptor signalling appears more nuanced. Pharmacological activation of D2/D3 receptors has been reported to enhance proliferation and differentiation in a region- and agonist-dependent manner in adult mice (Salvi et al., 2016), whereas genetic or lesion-based models implicate D2 signalling in neuroprotection or homeostatic regulation rather than direct pro-neurogenic effects (Park and Enikolopov, 2010; Dunleavy et al., 2013).

Understanding dopaminergic regulation of AHN is particularly relevant given dopamine’s involvement in neuropsychiatric and neurodegenerative disorders. For example, schizophrenia involves hyperdopaminergic signalling alongside reduced hippocampal volume and disrupted AHN (Brisch et al., 2014; Arnold et al., 2015). These observations suggest that dopaminergic excess can disrupt neurogenic homeostasis, highlighting the need for human-relevant models (Kasahara et al., 2022).

Despite growing interest in dopamine–neurogenesis interactions, most studies rely on rodent models whose neurogenic dynamics and receptor expression profiles may not fully reflect human physiology (Boldrini et al., 2018; Kempermann et al., 2018). Human post-mortem studies face technical challenges related to tissue quality, marker specificity, and post-mortem delay (Sorrells et al., 2018; Moreno-Jiménez et al., 2019). Consequently, human-based *in vitro* models provide a platform to directly assess dopaminergic effects on discrete stages of neurogenesis under controlled conditions.

Therefore, this study investigated the dose-dependent effects of dopamine on adult hippocampal neurogenesis using a human hippocampal progenitor cell model. Specifically, we examined how increasing concentrations of dopamine influence key neurogenic processes, including progenitor proliferation, cell survival, and neuronal differentiation. We also assessed whether these effects are modulated by dopamine receptor activity, focusing on the D4 receptor, previously identified as the predominant dopamine receptor in this cell line. Complementary qPCR analyses evaluated the expression of genes involved in progenitor maintenance (*ASCL1*, *AXIN2*, *GSK3β*), cellular stress responses (*HMOX1*, *NQO1*), and cell survival/apoptotic regulation (*BCL2L1*), providing a molecular perspective on dopamine’s actions. Together, this work aims to clarify how dopaminergic signalling regulates human hippocampal progenitor behaviour and address key translational gaps.

## 2 Materials and methods

### 2.1 Cell line and culture conditions

All experiments were performed using the human hippocampal progenitor cell line HPC0A07/03 (ReNeuron Ltd., Surrey, United Kingdom), as previously characterised (Borsini et al., 2022; Du Preez et al., 2022; 2024). Cells were originally derived from hippocampal tissue from a 12-week-old female foetus, in accordance with United Kingdom and US ethical and legal guidelines. The cell line was conditionally immortalised via retroviral transduction with a tamoxifen-inducible c-mycERTAM construct, allowing controlled proliferation in the presence of 4-hydroxytamoxifen (4-OHT) and spontaneous differentiation upon its withdrawal. All experiments were performed using cells between passages 20–25, with passage number balanced across experimental conditions and in each biological replicate.

Cells were maintained in reduced modified medium (RMM; DMEM/F12-based) supplemented with epidermal growth factor (EGF), basic fibroblast growth factor (bFGF), and 4-OHT during proliferation (Supplementary Table S1). Cells were cultured in laminin-coated Nunclon™ Delta-treated flasks at 37 °C, 5% CO_2_, and passaged at ∼90–95% confluency using accutase.

### 2.2 Proliferation and differentiation assays

Hippocampal progenitor cells were seeded into laminin-coated 96-well plates at a density of 1.3 × 10^4^ cells per well in proliferation medium. Each condition was plated in triplicate (technical replicates). Biological replicates were independently passaged cultures prepared on separate days (n = 4).

**FIGURE 1:**
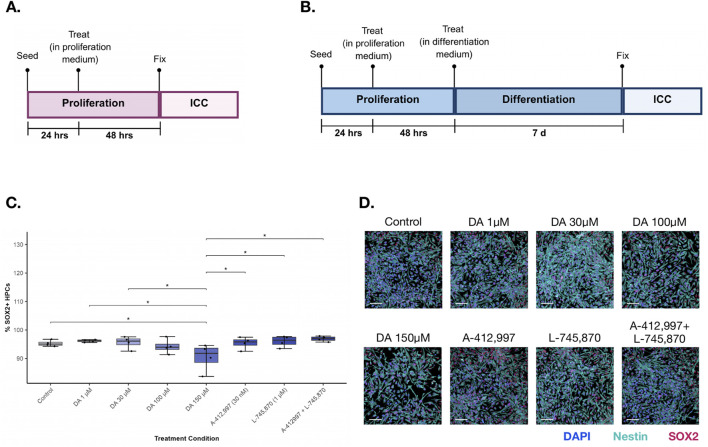
Effect of dopamine and receptor-targeting treatments on stemness marker during proliferation. **(A)** Proliferation timeline: Human hippocampal progenitor cells were maintained in proliferation medium and treated with dopamine or DRD4-targeting compounds for 42 h, after which cells were fixed for immunocytochemical analysis. **(B)** Differentiation timeline: Cells were switched to differentiation medium and treated with dopamine or DRD4-targeting compounds for 7 days prior to fixation and immunocytochemical analysis. **(C)** SOX2+ cell density across treatment groups. Two-way ANOVA revealed a significant reduction in ‘stemness’ through supraphysiological dopamine (150 µM) relative to several other conditions. Boxplots show the distribution of marker-positive cells per condition; boxes represent the interquartile range (IQR) with the median indicated by the horizontal line, and whiskers extending to 1.5× IQR. Individual data points represent biological replicates (n = 4; technical replicates = 3). Asterisks indicate statistical significance: *p < 0.05; **p < 0.01; ***p < 0.001. **(D)** Representative immunocytochemistry images of DAPI (blue), SOX2 (red) and Nestin (turquoise) across treatment conditions. Scale bar (bottom left) = 100 µm.

Cells were allowed to attach for 24 h prior to treatment. Dopamine-related compounds were then applied in fresh proliferation medium. Proliferation plates were fixed 48 h post-treatment (72 h post-seeding) using 4% paraformaldehyde (PFA) (Figure 1A). Differentiation plates received a second treatment at 72 h in differentiation medium lacking 4-OHT, EGF, and bFGF, and were maintained for a further 7 days before fixation (day 10 post-seeding) (Figure 1B). Fixation procedures were performed within a consistent daily time window (±1 h).

### 2.3 Pharmacological treatments

The control group consisted of cells maintained in proliferation or differentiation medium without any added solvent or pharmacological treatment. All pharmacological compounds were dissolved in distilled water and added to the culture medium.

#### 2.3.1 Dopamine hydrochloride

Dopamine hydrochloride (Sigma-Aldrich; MW = 189.64 g/mol) was prepared as a 10 mM stock solution in distilled water, sterile-filtered, aliquoted, and stored at −20 °C. Aliquots were thawed and diluted freshly on the day of each experiment immediately prior to use, with handling minimised and solutions protected from light by storing them in a dark box. Final concentrations of 1 μM, 30 μM, 100 μM, and 150 µM were used. The highest concentration (150 µM) exceeds physiological levels reported *in vivo* and is considered supraphysiological (Matt and Gaskill, 2020). While these procedures reduce degradation, dopamine autoxidation cannot be fully prevented at supraphysiological concentrations. This range was chosen to explore the maximal effect on progenitor cells.

#### 2.3.2 DRD4 agonist and antagonist

To investigate receptor-specific effects, a selective DRD4 agonist (A-412,997 dihydrochloride; TOCRIS) and antagonist (L-745,870 hydrochloride; Sigma-Aldrich) were used. A-412,997 was applied at 30 nM, and L-745,870 at 1 μM, based on reported receptor affinity and functional studies (Browman et al., 2005; Patel et al., 1997). A combined agonist + antagonist condition was included to assess receptor-level interactions. All compounds were prepared fresh on the day of use and thawed only once. DRD4 was selected because it is the most highly expressed dopamine receptor in this cell line, based on prior transcriptomic analyses (Supplementary Figure S1).

### 2.4 Immunocytochemistry

Cell number, progenitor identity, and apoptosis were assessed by immunocytochemistry using 4′,6-diamidino-2-phenylindole (DAPI), Nestin and SRY-box transcription factor 2 (SOX2), Ki67, and cleaved caspase-3 (CC3), respectively. Neuronal differentiation and maturation were evaluated using doublecortin (DCX) and microtubule-associated protein 2 (MAP2). Immunocytochemistry was performed following established protocols (Supplementary Table S2).

### 2.5 Imaging and quantification

Plates were imaged using the Opera Phenix High-Content Screening System (PerkinElmer). Quantification was performed using Harmony software. Marker-positive cells were expressed as a percentage of total DAPI-positive nuclei. DAPI density was quantified as the number of adherent nuclei per field using identical imaging, field selection, and segmentation parameters across all conditions and all plates. For differentiation markers, additional morphological parameters (including neurite length and Soma size) were quantified to assess neuronal maturation. Thresholds were defined using negative controls and held constant across experiments.

### 2.6 Quantitative PCR

Quantitative PCR (qPCR) was performed as an exploratory analysis of dopaminergic modulation. Cells were cultured under the conditions described above and seeded into 6-well plates at a density of 3 × 10^5^ cells per well. Cells were assigned to control (no dopamine), moderate (30 µM), or high/supraphysiological (150 µM) dopamine conditions (n = 3 per condition). For proliferation conditions, cells were treated for 24 h prior to RNA extraction (Figure 3A). For differentiation conditions, cells were maintained in proliferation medium for 24 h followed by differentiation medium for 4 days before RNA extraction (Figure 3B). Total RNA was extracted using the Zymo Research RNA extraction kit, and qPCR was performed using SYBR Green chemistry. Six genes related to neurogenesis, dopamine signalling, and cellular stress were analysed (Supplementary Table S3). Gene expression was normalised to *GUSB* and *LYNX* and calculated using the ΔΔCt method. Additional methodological details are provided in the Supplementary Material.

### 2.7 Statistical analysis

Statistical analyses were conducted using R (version 4.5.0). Technical replicates were averaged to yield one value per biological replicate. Data were assessed for normality using the Shapiro–Wilk test. Parametric data were analysed using two-way ANOVA with Treatment as the main factor and Batch included as a fixed factor to control for passage-related variability. Non-parametric data were analysed using Kruskal–Wallis tests. False discovery rate (FDR) correction was applied for multiple comparisons. Statistical significance was set at p < 0.05.

## 3 Results

### 3.1 Exposure to supraphysiological dopamine concentrations reduces hippocampal progenitor identity during proliferation

To assess effects of dopamine on cell viability and number during proliferation, DAPI-positive cell density was quantified across dopamine concentrations and receptor-targeting conditions. No significant effect of treatment was observed after accounting for batch effects (two-way ANOVA: F (7,23) = 2.13, p = 0.081). Consistent with this, Ki67 revealed no significant differences in proliferative activity across treatment groups (Kruskal–Wallis: χ^2^ (7) = 4.18, p = 0.759). Similarly, expression of cleaved caspase-3 (CC3), a marker of apoptosis, was not significantly altered by dopamine or pharmacological manipulation (two-way ANOVA: F (7,23) = 0.76, p = 0.624), although a significant batch effect was detected (F (1,23) = 64.19, p < 0.0001).

To examine whether dopamine influences progenitor identity, SOX2 and Nestin were assessed. Dopamine treatment had a significant effect on SOX2 expression (two-way ANOVA: F (7,23) = 2.69, p = 0.035; Figures 1C,D, Supplementary Figure S2). Post hoc comparisons indicated that the supraphysiological concentration of 150 µM dopamine significantly reduced SOX2 expression compared with the control condition (p = 0.0398), the 1 µM dopamine condition (p = 0.0294), the 30 µM dopamine condition (p = 0.0385), the agonist [A-412,997 dihydrochloride] (p = 0.0398), the antagonist-treated condition [L-745, 870 hydrochloride] (p = 0.0294), and the combined agonist and antagonist condition [A-412,997 dihydrochloride + L-745, 870 hydrochloride] (p = 0.023). No other pairwise comparisons reached statistical significance following FDR correction. In contrast, Nestin expression was not significantly affected by dopamine or receptor manipulation (Kruskal–Wallis: χ^2^ (7) = 6.30, p = 0.505).

### 3.2 Supraphysiological dopamine reduces cell survival and alters early neuronal morphology during differentiation

**FIGURE 2:**
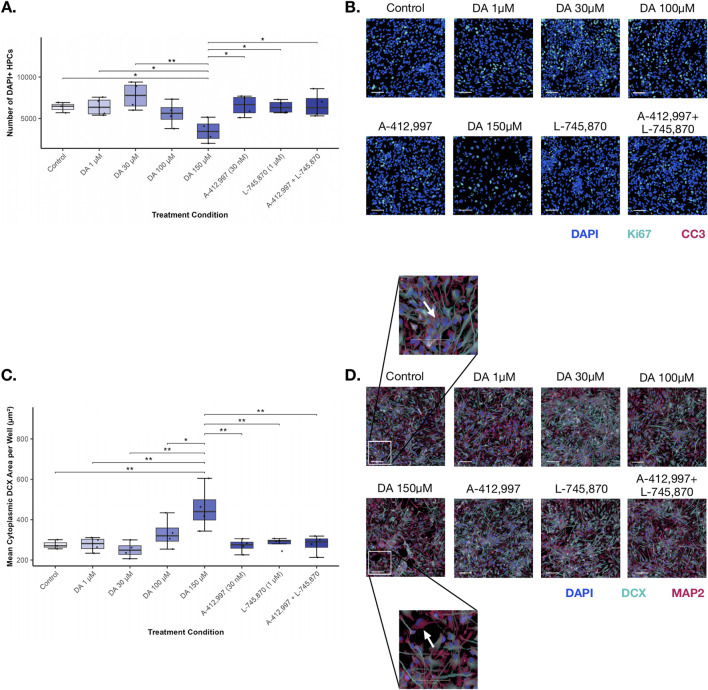
Effect of dopamine and receptor-targeting treatments on cell density and DCX+ area during differentiation. **(A)** DAPI+ cell density across treatment conditions. Two-way ANOVA revealed a significant effect of treatment, with 150 µM dopamine reducing cell density compared to control, physiological dopamine, and receptor-targeting conditions. Boxplots show median and interquartile range (IQR); whiskers extend to 1.5× IQR. Individual points represent biological replicates (n = 4; technical replicates = 3). Asterisks indicate statistical significance: *p < 0.05; **p < 0.01; ***p < 0.001. **(B)** Representative ICC images of DAPI (blue), Ki67 (turquoise), and CC3 (red). Scale bar = 100 µm. **(C)** DCX+ cytoplasmic area across treatment conditions. Two-way ANOVA with batch correction revealed a significant increase at 150 µM dopamine compared to all other conditions. Boxplots and points are shown as in **(A)**. **(D)** Representative ICC images of DAPI (blue), DCX (turquoise), and MAP2 (red). White squares indicate magnified inset regions, and white arrows highlight example cells with distinct cellular morphology. Scale bar = 100 µm.

During differentiation, dopamine treatment significantly affected DAPI-positive cell density (two-way ANOVA: F (7,22) = 5.08, p = 0.002; Figures 2A,B). A significant batch effect was also observed (F (1,22) = 10.91, p = 0.003). Post hoc analyses indicated that the supraphysiological dopamine concentration (150 µM) significantly reduced cell density compared with control, lower dopamine concentrations, and receptor-targeting conditions (all p < 0.05 after correction). No other treatment comparisons showed statistically significant differences.

Despite the reduction in overall cell density, dopamine exposure did not significantly affect proliferative activity during differentiation, as indicated by Ki67 expression (Kruskal–Wallis: χ^2^ (7) = 9.53, p = 0.218; Supplementary Figure S3). Similarly, CC3 expression was not significantly altered by treatment (two-way ANOVA: F (7,23) = 1.92, p = 0.112), although a batch effect was again observed (F (1,23) = 18.63, p < 0.001).

Expression of the immature neuronal marker DCX was not significantly affected by dopamine or receptor-targeting treatments (Kruskal–Wallis: χ^2^ (7) = 6.20, p = 0.517). Similarly, expression of the mature neuronal marker MAP2 showed no significant treatment effects (two-way ANOVA: F (7,22) = 1.02, p = 0.447).

Morphological analysis of DCX-positive cells revealed a significant effect of dopamine on cytoplasmic area during differentiation (two-way ANOVA: F (7,22) = 5.53, p < 0.001; Figures 2C,D). Post hoc comparisons demonstrated that the supraphysiological dopamine concentration significantly increased cytoplasmic area relative to all other treatment conditions (all p < 0.05). In contrast, mean neurite segment length was not significantly altered by treatment (Kruskal–Wallis: χ^2^ (7) = 4.05, p = 0.774).

### 3.3 Transcriptional changes associated with dopamine exposure

**FIGURE 3:**
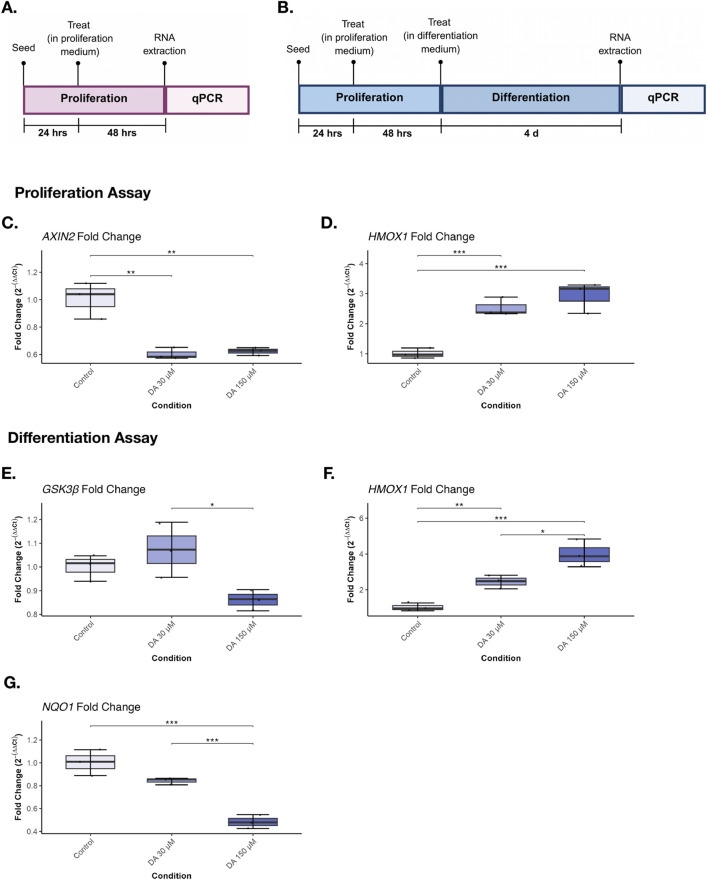
Dopamine alters expression of genes related to neurogenesis and cellular stress during proliferation and differentiation. **(A)** Proliferation timeline: Cells were treated under proliferation conditions for 42 h, after which RNA was extracted for gene expression analysis. **(B)** Differentiation timeline: Cells were differentiated for 4 days in the presence of dopamine or DRD4-targeting compounds prior to RNA extraction for qPCR analysis. Proliferation: **(C)** *AXIN2* expression was significantly reduced at 30 μM and 150 µM dopamine relative to control. **(D)** *HMOX1* expression was significantly increased at 30 μM and 150 µM dopamine. Differentiation: **(E)** *GSK3β* expression was significantly reduced at 150 µM dopamine compared with 30 µM. **(F)** *HMOX1* expression was significantly increased at 30 μM and 150 µM dopamine. **(G)** *NQO1* expression was significantly reduced at 150 µM dopamine relative to control and 30 µM conditions. Boxplots show median and interquartile range (IQR); whiskers extend to 1.5× IQR. Individual points represent biological replicates (n = 3) with technical replicates (n = 3). Asterisks indicate statistical significance: *p < 0.05; **p < 0.01; ***p < 0.001.

To investigate whether these effects were accompanied by changes in gene expression, we assessed transcriptional responses following dopamine exposure using qPCR. During the proliferation phase, dopamine exposure significantly altered expression of *AXIN2* (F (2,6) = 28.13, p < 0.001) and *HMOX1* (F (2,6) = 39.59, p < 0.001). Post hoc analyses indicated that both 30 μM and 150 µM dopamine significantly reduced AXIN2 expression relative to control, while significantly increasing *HMOX1* expression (Figures 3C,D). Expression of *ASCL1*, *GSK3β*, *BCL2L1*, and *NQO1* was not significantly altered (Supplementary Figure S4).

During differentiation, dopamine significantly affected expression of *GSK3β* (F (2,6) = 6.27, p = 0.034), *HMOX1* (F (2,6) = 39.69, p < 0.001), and NQO1 (F (2,6) = 43.78, p < 0.001). Post hoc analysis indicated that supraphysiological dopamine (150 µM) reduced *GSK3β* expression compared with the 30 µM condition, increased *HMOX1*, and decreased *NQO1* relative to control and moderate dopamine concentrations (Figures 3E–G). Expression of *AXIN2*, *ASCL1*, and *BCL2L1* did not remain significant following *post hoc* correction (Supplementary Figure S4).

## 4 Discussion

This study investigated the dose-dependent effects of dopamine on human hippocampal progenitor cells, aiming to determine its role in regulating adult hippocampal neurogenesis. During proliferation, supraphysiological dopamine (150 µM) significantly reduced SOX2 expression, putatively affecting neural stem cell identity. During differentiation, the same concentration significantly reduced DAPI+ cell number, indicating decreased cell survival. High dopamine also increased the cytoplasmic area of DCX+ cells, reflecting altered early neuronal morphology. No significant effects of dopamine were observed in proliferation (Ki67), apoptosis (CC3), progenitor identity (Nestin), early neuronal differentiation (DCX), or neuronal maturation (MAP2). Additionally, pharmacological manipulation of the D4 receptor had no measurable impact. Complementary qPCR analyses revealed changes in genes associated with oxidative stress (*HMOX1*, *NQO1*) and neurogenic signalling (*AXIN2*, *GSK3β*) under high dopamine exposure.

Exposure to supraphysiological dopamine significantly reduced DAPI+ cell density during differentiation, suggesting impaired cell survival in human hippocampal progenitor cells. Dopamine readily auto-oxidises, generating reactive oxygen species (ROS) and dopamine-quinones that disrupt mitochondrial and redox homeostasis (Hastings, 2009; Zhou et al., 2023). Consistent with ROS generation, the stress-inducible gene *HMOX1* increased while the antioxidant gene *NQO1* decreased, supporting a molecular stress response underlying reduced cell survival (Ma, 2013; Wu et al., 2021; Domanskyi and Parlato, 2022). This reduction in cell density was restricted to the differentiation phase, highlighting stage-specific vulnerability, as progenitors shift toward oxidative phosphorylation and become increasingly reliant on mitochondrial integrity (Garone et al., 2024). These early reductions in survival, together with disrupted progenitor identity and altered neuronal morphology, suggest that high dopamine exposure may compromise the generation of mature hippocampal neurons.

During proliferation, supraphysiological dopamine compromises neural progenitor identity, as shown by reduced SOX2 expression. Importantly, Nestin expression remains unchanged, reflecting selective modulation of SOX2-associated transcription rather than global loss of progenitor identity. Progenitor maintenance in the adult hippocampus is tightly regulated by intracellular signalling pathways, particularly Wnt/β-catenin signalling, which promotes neural stem and progenitor cell self-renewal and sustains SOX2 expression (Austin et al., 2021; Blassberg et al., 2022). SOX2 is essential for NSPC self-renewal and repression of premature differentiation, and even transient reductions in SOX2 impair long-term neurogenic capacity (Amador-Arjona et al., 2015; Pagin et al., 2021; Tsaytler et al., 2024). This effect was accompanied by reduced expression of *AXIN2*, a canonical transcriptional target of Wnt/β-catenin signalling, indicating that high dopamine diminishes Wnt pathway activity, which likely contributes to the observed reduction in SOX2. These results collectively suggest that high dopamine may impair Wnt-dependent transcriptional programmes sustaining progenitor identity, with SOX2 loss serving as an early indicator of this disruption.

During differentiation, supraphysiological dopamine increased the cytoplasmic area of DCX+ cells without affecting neurite length, suggesting a potential change in early neuronal morphology. DCX is a microtubule-associated protein that regulates cytoskeletal dynamics in immature neurons (Dema et al., 2024). Parallel to this, *GSK3β* expression was reduced at 150 µM dopamine compared with moderate concentrations. *GSK3β* is a key regulator of microtubule stability and cytoskeletal organization, and its downregulation may contribute to the observed enlargement of DCX+ cell bodies by altering microtubule dynamics and intracellular structural integrity (Choi et al., 2020). These changes are consistent with dopamine-induced oxidative stress, which can further influence cytoskeletal remodelling in immature neurons (Gardiner et al., 2013; Chen et al., 2022). Overall, high dopamine appears to alter cytoskeletal organization in early neuronal progenitors through combined effects on oxidative stress pathways and Wnt/cytoskeletal regulators such as GSK3β.

Despite morphological changes, no significant differences were observed in other proliferation or differentiation markers, including Ki67, Nestin, MAP2, and CC3. This lack of change, particularly in CC3, was unexpected given that numerous studies have identified dopamine-induced apoptosis in neural cells (Cheng et al., 1996; Jones et al., 2000; Porat et al., 2001). For instance, Cheng et al. (1996) demonstrated that exposure of primary striatal neurons to 30–300 μM dopamine or L-DOPA resulted in dose-dependent apoptotic cell death, suggesting that dopamine’s cytotoxic potential should be observable through caspase activation. In the present study, the lack of CC3 activation may reflect delayed late-stage apoptosis beyond the 7-day assay window, involvement of alternative non–caspase-dependent mechanisms, or cell loss due to detachment, which would not be captured by CC3 alone.

In the case of Ki67, while no statistically significant differences emerged, a non-significant dose-dependent trend was observed, with a peak at 30 µM dopamine (Supplementary Figure S3). This trend mirrors findings by Takamura et al. (2014), who reported that low to moderate doses of dopamine (30–100 µM) enhance hippocampal neural progenitor proliferation via D1 receptor signalling and Wnt/β-catenin pathway activation. The concurrent non-significant increase in Ki67 and MAP2 at 30 µM may suggest a threshold beyond which dopamine transitions from supporting progenitor function to inducing stress-related effects.

Interestingly, there was a reduction in *AXIN2* expression during proliferation observed at both moderate and supraphysiological dopamine concentrations. This further supports impairment of Wnt/β-catenin signalling as an early molecular response to dopamine exposure. As a canonical Wnt target gene, *AXIN2* is a sensitive readout of pathway activity in neural progenitors (Jho et al., 2002). Importantly, changes in Wnt-dependent transcription can precede measurable changes in cell-cycle markers such as Ki67, indicating early molecular sensitivity to dopaminergic modulation (Ding et al., 2017). In human neural progenitor systems, this early transcriptional reprogramming may not immediately translate into detectable proliferative changes due to tightly regulated signalling thresholds (Kuwabara et al., 2009; Zhang et al., 2013).

Together, these findings highlight the complex and stage-specific influence of dopamine on human hippocampal progenitors. While high dopamine alters stemness and early neuronal morphology, markers of proliferation and apoptosis remain largely unchanged, possibly reflecting delayed or buffered responses at this stage of neurogenesis. This emphasises the importance of considering both molecular and morphological readouts when assessing dopaminergic modulation of neurogenesis.

Across all measured markers, neither the DRD4-selective agonist nor antagonist produced significant effects. DRD4 was selected based on single-cell RNA sequencing showing it as the most abundantly expressed dopamine receptor in HPC0A07/03 cells; however, high transcript levels do not necessarily reflect protein expression or functional engagement. Moreover, as transcriptional analyses were limited to dopamine-treated conditions, receptor-specific downstream effects of DRD4-selective manipulation were not assessed. Consequently, conclusions regarding functional engagement of DRD4 under the present assay conditions are limited. DRD4 has relatively low affinity compared to other receptor subtypes, and D1-like receptors are more commonly implicated in regulating neural progenitor proliferation and differentiation (Popolo et al., 2004). These findings suggest that the effects of 150 µM dopamine may be mediated by other receptor pathways, such as DRD1-, DRD2-, or non-dopaminergic receptors, as well as receptor-independent mechanisms like oxidative stress. Additionally, dopamine autoxidation may further contribute, making it difficult to distinguish receptor-driven effects from oxidative-related mechanisms.

DRD4 signalling in neural progenitors remains poorly characterised. Although it can modulate cAMP and G-protein-coupled pathways in other systems, evidence linking it to canonical neurogenic cascades, such as Wnt/β-catenin or PI3K/Akt, is limited (Mishra et al., 2019; Zhang et al., 2021; Zhou et al., 2024). It may also regulate aspects of progenitor physiology not captured by the current markers, including metabolism, or synaptic priming (Ptáček et al., 2011; Gilsbach et al., 2012). Dopaminergic signalling often involves receptor interactions, so selectively targeting a single receptor may be insufficient to produce measurable effects. Overall, these results highlight the limitations of relying solely on transcriptomic data to infer receptor function and underscore the complexity of dopamine signalling in human hippocampal stem cells.

This study used a human hippocampal stem cell model to investigate dopamine’s effects on adult hippocampal neurogenesis. Compared with animal models, this approach avoids confounding factors such as species-specific differences in receptor expression, systemic physiological responses, and behavioural influences like stress or physical activity (Farmand et al., 2025; Shin et al., 2024). Consequently, it provides insight into the direct effects of dopamine on human neural progenitors. However, *in vitro* models cannot fully replicate the complex neuromodulatory environment of the hippocampus, where dopaminergic signalling is influenced by tonic versus phasic release, interactions with other neurotransmitters, receptor localization, and network activity (Di Giovanni, 2010; Sheynikhovich et al., 2011). Additionally, although derived from a female foetus, HPC0A07/03 cells lack sex hormone-dependent regulation, and potential sex-specific dopaminergic effects cannot be assessed.

A key strength of the study is its focus on early stages of neurogenesis, allowing detailed analysis of initial cellular responses. However, only early proliferation and differentiation markers were assessed, and astrocytic differentiation (e.g., GFAP) was not evaluated, so downstream effects such as long-term maturation, glial differentiation, or functional outcomes remain unclear. Future studies incorporating extended differentiation, astrocytic markers, and functional assays could clarify long-term neurogenic outcomes.

**FIGURE 4:**
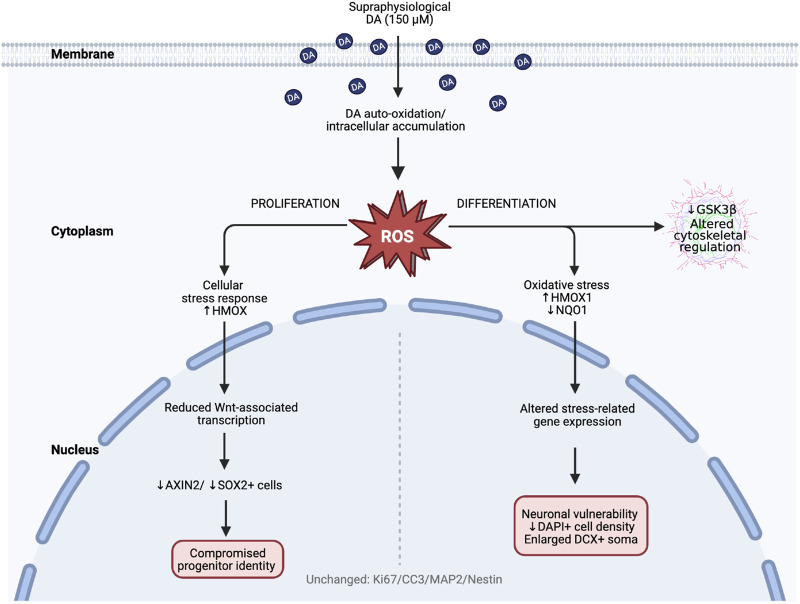
Proposed mechanistic model of dopamine-induced stress across hippocampal proliferation and differentiation stages. Schematic summary of molecular and cellular findings following exposure of human hippocampal progenitor cells to supraphysiological dopamine (150 µM). Dopamine autoxidation increases intracellular ROS and activates a cellular stress response. During proliferation (left), dopamine reduces AXIN2 and SOX2 expression, suggestive of impaired Wnt/β-catenin signalling and compromised progenitor identity. During differentiation (right), oxidative stress is associated with increased HMOX1, reduced NQO1 and GSK3β expression, decreased DAPI+ cell density, and enlargement of DCX+ neuronal Soma. Created in BioRender. Thuret (2026) https://BioRender.com/zqn8uxl.

The dose-response design, including a supraphysiological dopamine concentration (150 µM), may induce receptor-independent oxidative stress via autoxidation, providing a relevant *in vitro* model of redox imbalance observed in neurological disorders. Complementary qPCR analyses revealed early transcriptional changes in oxidative stress, Wnt/β-catenin signalling, and cell survival, which likely precede detectable changes in proliferation or apoptosis. These molecular disruptions may underlie the observed reductions in SOX2 expression and altered DCX+ morphology, highlighting the sensitivity of progenitor identity and early neuronal structure to dopaminergic stress (Figure 4). However, since direct ROS measurements or antioxidant rescue experiments were not performed, oxidative stress remains a plausible but unconfirmed limiting mechanism. While the limited gene panel and short assay window constrain detection of longer-term effects, the findings suggest that supraphysiological dopamine triggers early molecular and structural changes in hippocampal progenitors. These mechanisms may be particularly relevant to hyperdopaminergic conditions, including schizophrenia, which are associated with hippocampal atrophy, elevated ROS, and impaired neurogenesis (Burbulla et al., 2017; Kesby et al., 2018; Masato et al., 2021; Murray et al., 2021; Sasabayashi et al., 2021).

Future studies should investigate the receptor-specific mechanisms underlying these effects, particularly DRD1-mediated signalling, and explore how oxidative stress and mitochondrial dysfunction interact with key neurogenic pathways such as Wnt/β-catenin. Extending differentiation timelines, incorporating functional assays, and performing detailed morphological analyses could clarify how these early molecular and structural perturbations translate into long-term deficits in neuronal maturation, integration, and hippocampal function. Such work would help link dopaminergic dysregulation to cognitive and neuropsychiatric outcomes, providing an understanding of disease-relevant vulnerabilities.

## 5 Conclusion

Our study shows that dopamine exerts dose-dependent effects on human hippocampal progenitor cells. Supraphysiological concentrations impair cell survival, reduce SOX2 expression, and alter early neuronal morphology, potentially via oxidative stress–mediated mechanisms. Low-to-moderate dopamine levels did not significantly enhance neurogenesis markers, though subtle trends suggest a narrow window in which dopamine may support proliferation and differentiation. DRD4-selective modulation had no observable effects, implicating alternative receptors or receptor-independent mechanisms. Overall, this work advances understanding of dopaminergic regulation in a human-relevant model of adult hippocampal neurogenesis and highlights the importance of tightly regulated dopamine signalling for progenitor health and neurogenic potential.

## Data Availability

The original contributions presented in the study are included in the article/Supplementary Material, further inquiries can be directed to the corresponding author.
