# Supplementary material for: High dopamine impairs early neuronal identity and morphology in human hippocampal progenitor cells
Source: Front Cell Dev Biol. 2026 Mar 30;14:1783035. doi: 10.3389/fcell.2026.1783035 (PMC13070916; doi:10.3389/fcell.2026.1783035)
Supplement: Supplementary file 1 [file DataSheet1.pdf]

Supplementary Material:

Table S1. Composition of the media used for cell culture with corresponding concentrations.

Formula of Reduced Modified Media (RMM)	
**Material Names**	**Final Conc.**
DMEM F12: Dulbecco's modified eagle's medium nutrient mixture f-12 ham	–
Human Albumin Solution	0.03%
Apo-Transferrin, human	100 µg/ml
Putrescine DiHCl	16.2 µg/ml
Insulin, human recombinant	5 µg/ml
Progesterone	60 ng/ml
L-glutamine	2 mM
Sodium Selenite	40 ng/ml
HEPES	15 mM
**Additions for Proliferation Media**	
4-OHT: 4-hydroxytamoxifen	100 nM
EGF: Epidermal Growth Factor	20 ng/ml
bFGF: Basic Fibroblast Growth Factor	10 ng/ml

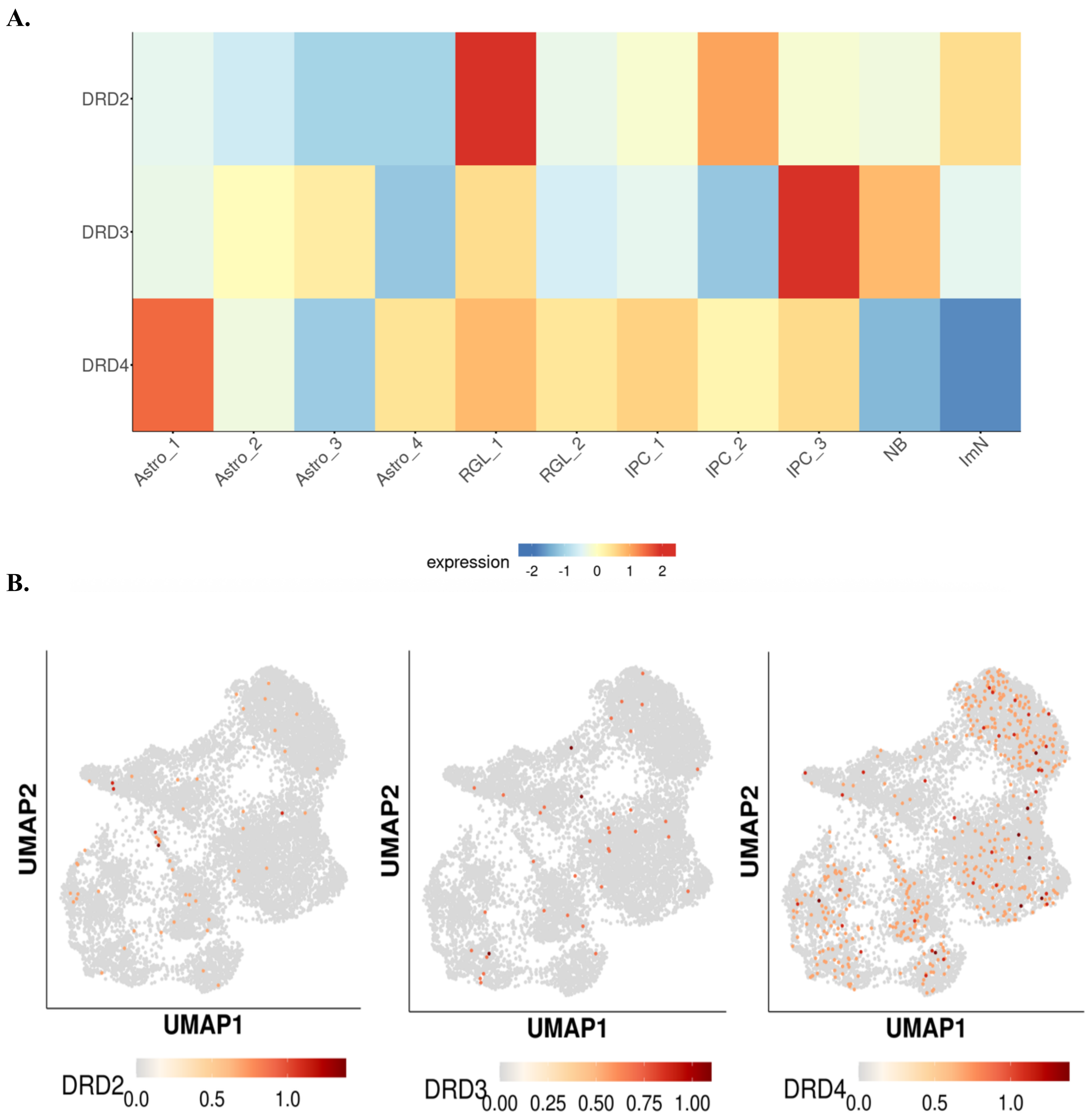

Figure S1. Single-cell RNA sequencing analysis of dopamine receptor expression in the human hippocampal stem cell line, HPC03A/07 (data provided by Daniel Rock, Thuret Lab). (A) Heatmap showing gene expression levels of dopamine receptor subtypes DRD2, DRD3, and DRD4 across different cell types within the cell line. (B) UMAP plots displaying individual expression patterns of DRD2, DRD3, and DRD4 receptors, highlighting DRD4 as the predominant subtype expressed.

Immunocytochemistry Protocol

After fixation, cells were permeabilised and blocked using 0.3% Triton X-100 in PBS with 0.05% sodium azide and 5% normal donkey serum (NDS) for 1 hour at room temperature. Primary antibodies (see **Table S2**) were applied at 30 µL per well and incubated overnight at 4°C. Markers used in the proliferation assay included Ki67 (proliferation), Cleaved Caspase-3 (CC3) (apoptosis), Nestin (progenitor marker), and SOX2 (pluripotency marker). In the differentiation assay, Ki67 and CC3 were again included, along with Doublecortin (DCX) (immature neurons) and microtubule-associated protein 2 (MAP2) (mature neurons).

After primary incubation, wells were washed and re-blocked for 30 minutes before secondary antibodies were applied: Donkey anti-Rabbit Alexa Fluor 555 (A31572) and Donkey anti-Mouse Alexa Fluor 488 (A21202), both at 1:500 dilution, incubated for 2 hours at room temperature in the dark. 4′,6-diamidino-2-phenylindole (DAPI), a nuclear counterstain, was added for 5 minutes post-secondary incubation. Final PBS washes were performed, and plates were stored at 4°C in the dark in PBS with 0.05% sodium azide.

Table S2. Primary antibodies used for immunocytochemical analysis. Details for each antibody include the target antigen, assay type, supplier and catalogue number, working dilution, and the functional role of the marker.

Primary Antibodies				
**Antibody**	**Assay Type**	**Company; Catalogue No.**	**Dilution Factor**	**Function**
Mouse anti-Ki67	Proliferation & differentiation	CellSignalling; 9949	1:800	Proliferation marker
Rabbit anti-CC3	Proliferation & differentiation	Invitrogen; PA5-114687	1:1000	Apoptosis marker
Rabbit anti-SOX2	Proliferation	Chemicon; ab5603	1:1000	Neural stem cell marker (transcription factor)
Mouse anti-Nestin	Proliferation	Chemicon; mab5326	1:1000	Neural stem cell marker (intermediate filament)
Rabbit anti-DCX	Differentiation	Abcam; ab18723	1:500	Neuroblast marker
Mouse anti-MAP2	Differentiation	Abcam; ab11267	1:500	Neuron marker

<u>Quantitative PCR (qPCR)</u>

Quantitative PCR was performed to assess transcriptional changes associated with dopaminergic modulation. Cells were cultured and treated as described in the main Methods and seeded into 6-well plates at a density of 3×10^5 cells per well. Dopamine was applied at control, moderate (30 µM), or high (150 µM) concentrations, with three independent biological replicates per condition. For proliferation, cells were treated for 24 hours prior to RNA extraction. For differentiation, cells were maintained in proliferation medium for 24 hours followed by 4 days in differentiation medium before RNA extraction. This time point was selected to capture early transcriptional changes during neuronal differentiation; longer differentiation periods were reserved for protein-level analyses.

RNA Extraction and cDNA Synthesis:

Total RNA was extracted using the Zymo Research RNA extraction kit according to the manufacturer's instructions. RNA concentration and purity were assessed using a NanoDrop spectrophotometer. cDNA was synthesised from total RNA using the Thermo Fisher High-Capacity cDNA Reverse Transcription Kit, following the manufacturer's protocol.

qPCR Conditions:

qPCR was performed using SYBR Green chemistry on a QuantStudio 7 with the following cycling parameters: initial denaturation at 95 °C for 2 min, followed by 40 cycles of denaturation at 95 °C for 5 s, annealing at 60 °C for 30 s, and extension at 72 °C for 30 s. Melt curve analysis was performed from 60–95 °C to confirm amplification specificity. All reactions were run in technical triplicates.

Data Analysis:

Ct values were determined using the instrument software. Relative gene expression was calculated using the ΔΔCt method, normalised to the geometric mean of reference genes *GUSB* and *LYNX1*, and expressed relative to control.

Table S3. Genes assessed to complement immunocytochemistry, including gene name, functional relevance, and corresponding forward and reverse primer sequences.

Genes Assessed			
**Gene**	**Function/ Relevance**	**Forward Primer (5'→ 3')**	**Reverse Primer (5'→ 3')**
ASCL1	Progenitor maintenance	AGAAACAAGAAGGCGCCAGCGG	TTTTTCTCCTCCCTGCACGCCG
AXIN2	Wnt/β-catenin signalling	TCCAAGGCTCCGGAAACCATGC	GCCTTTCCCATTGCGTTTGGGC
GSK3β	Wnt/ cytoskeletal regulation	TTGAGTGATGGCCGCCTTTCCC	ACAACCATGGGCTCAGCTCTGC
HMOX1	Oxidative stress response	TGAATGCAGGCATGCTGGCTCC	TGAAAGCCTTCAGTGCCCACGG
NQO1	Antioxidant defence	TTCACCATGTTGGCCAGGCTGG	AGATTGCAAGGGCCAGGTGTGG
BCL2L1	Cell survival/ apoptosis	AACCAACAAGACCCAGCACCGC	TTCTGAAACGTCACCTGCCCCC
GUSB	Reference gene	ATGTGGTCTGTGGCCAACGAGC	TGCTCACAAAGGTCACAGGCCG
LYNX1	Reference gene	TCAGCCCAACACGCACCAATCC	TGGCACCAAAAGGTCTCACGCC

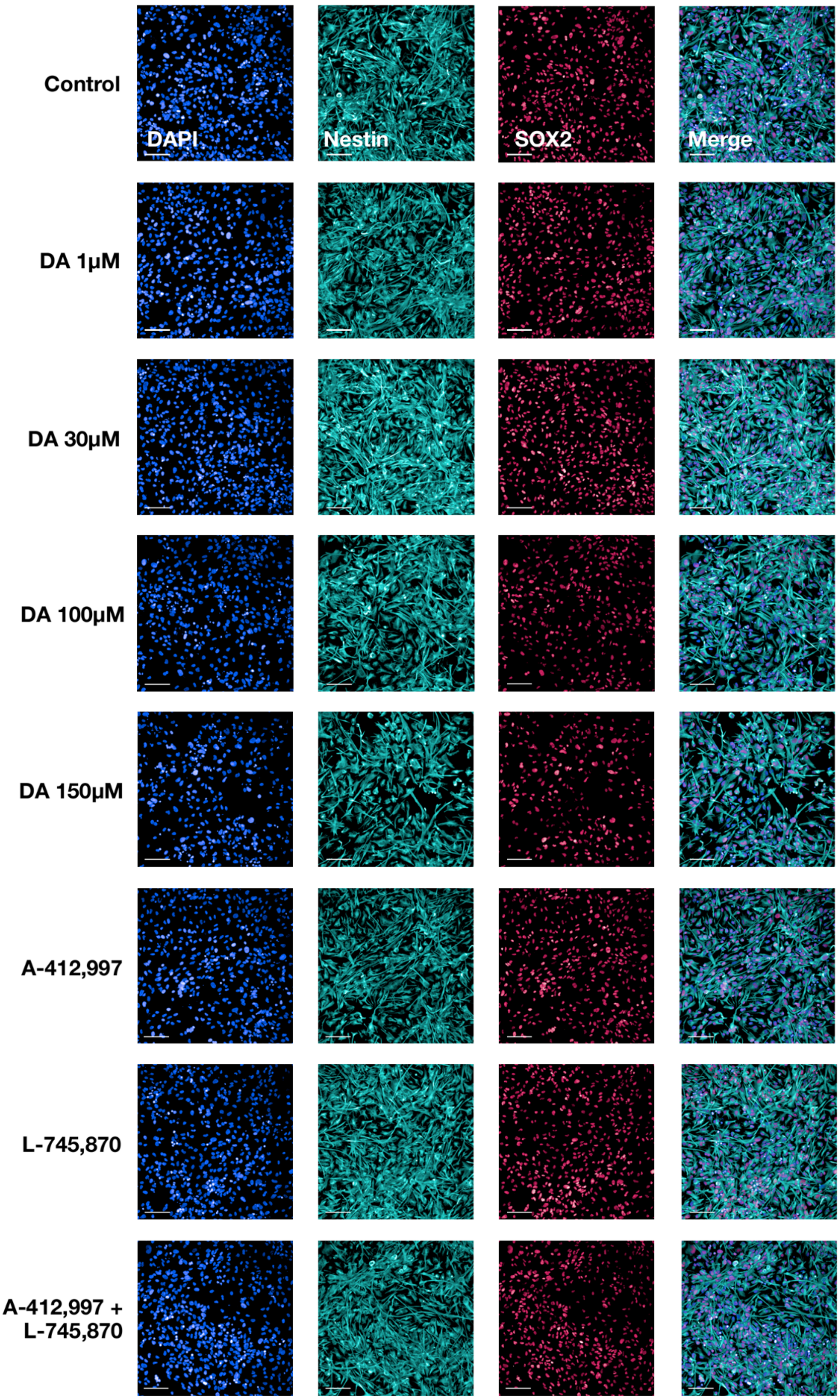

Figure S2. Individual fluorescence channels corresponding to Figure 1. Representative immunocytochemistry images of human hippocampal progenitor cells during proliferation following dopamine and receptor-targeting treatments. Images display individual staining channels for DAPI (blue), Nestin (turquoise), and SOX2 (red), shown separately to clarify marker localization. Scale bar = 100 µm.

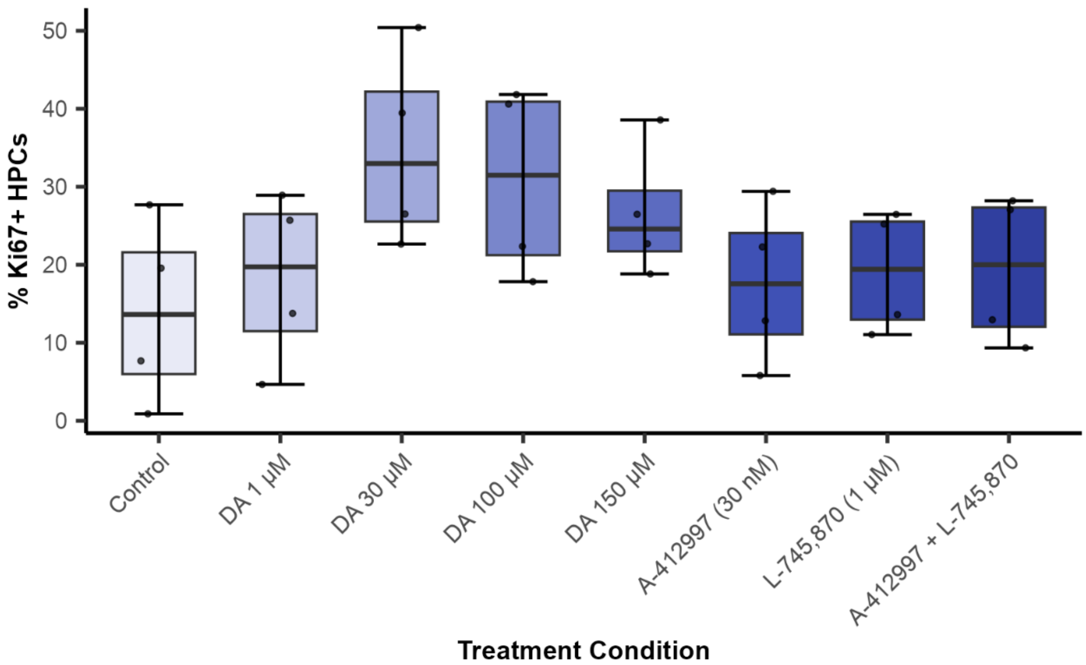

Figure S3. Assessment of proliferation marker during the differentiation phase following dopamine and receptor-targeting treatments. Graph shows Ki67+ cell density across treatment groups. No significant differences in proliferation were observed across conditions, as determined by Kruskal–Wallis test. Boxplots show the distribution of marker-positive cells per condition; boxes represent the interquartile range (IQR) with the median indicated by the horizontal line, and whiskers extending to 1.5× IQR. Individual data points represent biological replicates (n = 4; technical replicates = 3).

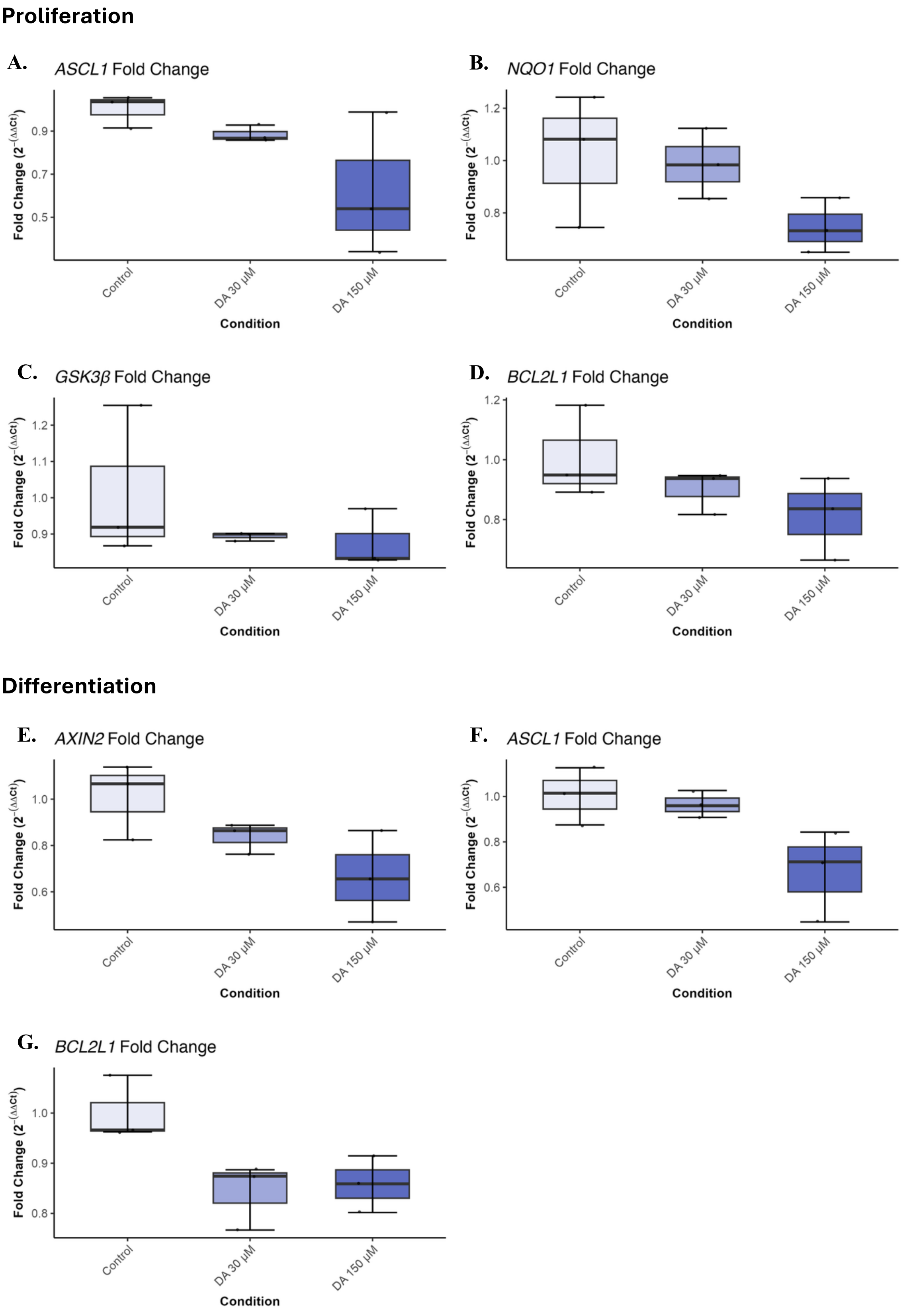

Figure S4. Expression of additional neurogenesis- and survival-related genes during proliferation and differentiation following dopamine exposure. Proliferation: (A) *ASCL1*, (B) *NQO1*, (C) *GSK3β*, and (D) *BCL2L1* expression showed no significant differences across dopamine concentrations. Differentiation: (E) *AXIN2*, (F) *ASCL1*, and (G) *BCL2L1* expression were not significantly altered by dopamine treatment. Boxplots show median and interquartile range (IQR);

whiskers extend to 1.5× IQR. Individual points represent biological replicates (n = 3) with technical replicates (n = 3). No comparisons reached statistical significance.
